# Transcriptional control of blood cell emergence

**DOI:** 10.1002/1873-3468.13585

**Published:** 2019-08-31

**Authors:** Sara Menegatti, Marcel de Kruijf, Eva Garcia‐Alegria, Georges Lacaud, Valerie Kouskoff

**Affiliations:** ^1^ Developmental Haematopoiesis Group Faculty of Biology, Medicine and Health the University of Manchester UK; ^2^ Cancer Research UK Stem Cell Biology Group Cancer Research UK Manchester Institute The University of Manchester Macclesfield UK

**Keywords:** embryonic haematopoiesis, haemogenic endothelium, transcription network

## Abstract

The haematopoietic system is established during embryonic life through a series of developmental steps that culminates with the generation of haematopoietic stem cells. Characterisation of the transcriptional network that regulates blood cell emergence has led to the identification of transcription factors essential for this process. Among the many factors wired within this complex regulatory network, ETV2, SCL and RUNX1 are the central components. All three factors are absolutely required for blood cell generation, each one controlling a precise step of specification from the mesoderm germ layer to fully functional blood progenitors. Insight into the transcriptional control of blood cell emergence has been used for devising protocols to generate blood cells *de novo*, either through reprogramming of somatic cells or through forward programming of pluripotent stem cells. Interestingly, the physiological process of blood cell generation and its laboratory‐engineered counterpart have very little in common.

## Graphical abstract

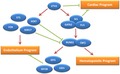

## Abbreviations

**EHT**, endothelium‐to‐haematopoietic transition

**ESCs**, embryonic stem cells

**HE**, haemogenic endothelium

**HSCs**, haematopoietic stem cells

**HSPCs**, haematopoietic stem and progenitor cells

In adult organisms, the haematopoietic system is sustained throughout life by a pool of haematopoietic stem cells (HSCs) that resides in the complex microenvironment of the bone marrow 1. This pool of HSCs maintains itself through self‐renewal; it is not clear, however, whether HSCs are slowly cycling, or mostly quiescent with only a subset of cycling HSCs 2. Under homeostatic conditions, HSCs give rise to all blood cells of the erythroid, myeloid and lymphoid lineages through a discreet series of differentiation steps leading to fully mature blood cells. This equilibrium between self‐renewal and differentiation is carefully controlled by the bone marrow microenvironment and is critical to the sustained production of all blood cells. Any alteration in this balancing act such as in certain haematological disorders leads to bone marrow failure, a condition in which HSCs become exhausted 3. Left untreated, this is unfortunately a terminal illness as there is no *de novo* generation of HSCs in adult organisms. HSCs are only generated *de novo* during embryonic development when the haematopoietic system is first established.

## Embryonic emergence of the haematopoietic system

During embryonic development, the haematopoietic system emerges in sequential waves, each characterised by its specific timing, location and type of progenitors generated. Soon after gastrulation, mesoderm progenitors within the primitive streak migrate to the developing yolk sac to form mesodermal masses that, by E7.5 in the mouse embryo, form blood islands composed of primitive erythrocytes surrounded by endothelial cells 4. This first wave of haematopoiesis also gives rise to megakaryocytes 5, macrophages and tissue‐resident macrophages such as microglia of the brain 6. This first wave is closely followed by a second wave of precursor emergence within the yolk sac vasculature of E8.5 mouse embryos. At this stage, erythro‐myeloid progenitors are produced which, upon maturation, generate definitive erythrocytes, and all types of myeloid cells 7. The generation of lymphoid progenitors shortly follows and occurs both within the yolk sac and the embryo proper by E9.0–9.5 8, 9. The first HSCs, capable of adult engraftment, are only detected by E10.5, emerging from the major arteries of the developing embryo 10, 11. HSCs are found in the yolk sac and placenta later on, but it is still not clear whether they arise autonomously within those sites or if they are transported there from their site of emergence *via* the circulation 12, 13. Newly formed HSCs migrate to the liver where considerable expansion takes place 14; from E14.5 onwards, HSCs start colonising the spleen, and ultimately the bone marrow, where they will reside thereafter 15.

## Endothelial origin of all blood cells

Seminal observations dating back from the early 19th century suggested a very close lineage relationship between endothelium and blood cells during embryonic development, coining terms such as haematoblast 16, haemocytoblast 17 or haemangioblast 18. The endothelial origin of blood cells was formally demonstrated decades later with the advance of experimental approaches allowing cellular marking 19 and lineage tracing 20. All blood cells are derived from FLK1‐expressing mesoderm 21 through endothelium intermediates; whether these FLK1 mesoderm precursors can be termed haemangioblast remains a matter of debate discussed elsewhere 22. Endothelium giving rise to blood cells are defined as haemogenic endothelium (HE) and are found at all sites of blood cell emergence. Through a process of endothelium‐to‐haematopoietic transition (EHT), HE subsets were shown to generate primitive erythrocytes 23, erythro‐myeloid progenitors 24, B lymphocytes 9 and HSCs 25. This EHT process is akin to the well‐characterised epithelial to mesenchyme transition and entails a differentiation process involving dramatic morphological and transcriptional changes. In the literature, the definition of HE is often associated with the potential to generate both endothelial and haematopoietic cells. However, the current lack of specific markers hinders the distinction between HE and non‐HE. Thus, at present, it is not possible to determine, and therefore to claim, that HE generates endothelium. Rather, HE can only be identified retrospectively, once it has produced blood cells.

## Transcriptional control of mesoderm specification to endothelium and haemogenic endothelium

### ETV2

**Figure 1:**
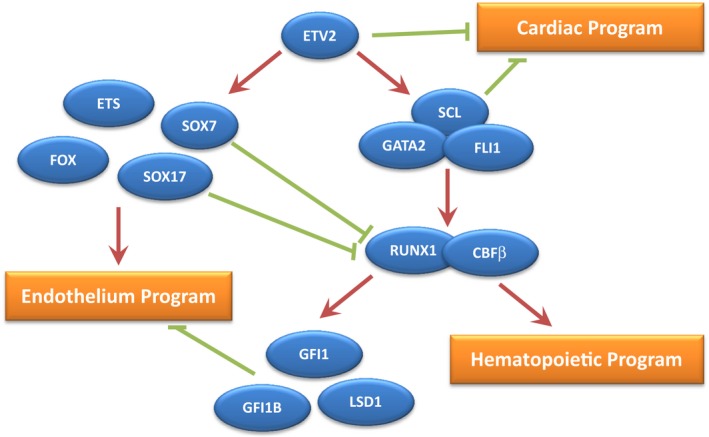
Schematic representation of the transcription factor network controlling endothelium and haematopoietic specification. Transcription factors are depicted in blue, positive activities are depicted in red and repressive activities in green.

Once mesoderm is formed, the first known transcription factor regulating further specification towards haematopoiesis is the ETS family member ETV2. This ETS transcription factor is expressed between embryonic day E6.5 and E9.5 in the mouse embryo, with an expression pattern primarily restricted to the yolk sac, where its expression marks all nascent endothelium 26. Remarkably, ETV2 deficiency leads to a complete absence of all blood cells and organised vasculature 27. However, the conditional deletion of ETV2 in FLK1‐expressing cells 28 or TIE2‐expressing cells 29 does not affect blood cell emergence or vasculature organisation. This suggests that ETV2 acts as a temporal switch for these lineages, during early embryonic development, at the onset of FLK1 expression. Analysis of the downstream targets of ETV2 implicated in these developmental processes established this transcription factor as a master regulator of both blood and endothelium programs (Fig. 1), regulating the expression of genes such as *Sox7*,* Scl* or *Gata2* 30, 31, 32. Using embryonic stem cells (ESCs) *in vitro* differentiation to study haematopoietic specification, Wareing *et al*. observed that the expression of SCL was sufficient to fully restore blood cell emergence in *Etv2* ^*−/−*^ cells 28, demonstrating the unique role of ETV2 in switching on the haematopoietic program *via* SCL; similar observations were made using the Zebrafish model system 33.

It is interesting to note that the function of ETV2 is not fully conserved across evolution: the Etsrp/ER71 Zebrafish homolog is required for vascular development and myeloid lineages but is dispensable for erythroid lineages 34 while the ER71 Xenopus homolog is only required for vascular development and does not control blood cell emergence 35. The work of Liu and colleagues suggests that FLI1, another member of the ETS family, orchestrates the earliest stage of mesoderm specification to the cardiovascular system in Xenopus and Zebrafish and that ETV2 acts downstream of FLI1 in these model organisms 36. In an *in vitro* mouse ESC model of blood cell emergence, the expression of FLI1 in ETV2^−/−^ FLK1 mesoderm was, however, unable to restore haematopoiesis 28, demonstrating that in the mouse system the function of these two ETS factors is not interchangeable. The reason behind the progressive changes in ETV2 and FLI1 functions during evolution remains to be determined.

### SCL

As discussed above, directly downstream of ETV2, the basic Helix‐loop‐helix transcription factor SCL (also known as TAL1) controls blood cell emergence (Fig. 1). SCL was originally identified at breakpoint of chromosomal translocations associated with the occurrence of leukaemias 37, 38 and was later shown, through knockout approaches, to be indispensable for the generation of all blood cells *in vivo* 39, 40 and *in vitro* 41, 42. In *Scl* ^*−/−*^ embryos, vascular development is not as dramatically affected as observed in *Etv2* ^*−/−*^ embryos, but SCL deficiency does lead to remodelling defects in the yolk sac vascular network 43, 44. Similar to ETV2, the expression of SCL is only temporally required for blood cell emergence as its conditional deletion in TIE2‐expressing cells does not affect blood cell emergence 45. In line with this restricted temporal requirement, *in vitro* studies have demonstrated the critical role of SCL for the formation of haemangioblast 46 and HE 47. As discussed elsewhere, SCL is also an important player in adult haematopoiesis and erythropoiesis 48.

SCL functions within multi‐factor complexes containing LMO2, E2A, LDB1, FLI1 and GATA2 49, 50. The genome‐wide analysis of SCL targets *via* chromatin immunoprecipitation has revealed a large number of downstream transcriptional targets, including many genes with known implication in both endothelial and haematopoietic cell fate, including many transcription factors such as *Sox7*,* Sox17*,* Gata1*,* Gata2*,* Erg*,* Fli1* or *Myb* 51, 52, 53. Among those, *Runx1* has been recurrently identified as a critical downstream transcriptional target of SCL at the onset of blood cell emergence 54, 55, 56.

## Cardiac versus haematopoietic fate

Both ETV2 and SCL are critical transcriptional activators of the haematopoietic and endothelial programs, and while they are actively promoting these cell fates, experimental evidence suggests that they are also actively repressing the cardiomyocyte fate (Fig. 1). In an ESCs differentiation system promoting cardiac, endothelial and haematopoietic lineage specification, the enforced expression of ETV2 was shown to inhibit the specification of cardiac mesoderm, which gives rise to cardiomyocytes and smooth muscle cells 57. This was shown to occur *via* inhibition of the Wnt signalling pathway. Similar findings were also observed in Zebrafish embryos, in which ETV2 deficiency led to increase formation of cardiomyocytes 58. Additional studies further established that the transcriptional inhibition of ETV2 expression was a necessary step for cardiac specification 59. In a similar line of investigation, several studies demonstrated the repressive role of SCL on cardiomyocyte specification through enhancer competitive occupancy 53, 60 and recruitment of Polycomb repressive complexes 52 to cardiomyocyte specific genes. It is not clear if both mechanisms of cardiomyocyte lineage repression function in concert or whether they are each active at different stages of the process. Single‐cell transcriptomic analysis of *Scl* ^−/−^ early embryos suggest that the primary role of SCL is blood specification and that cardiomyocyte specification might be a later event in cells unable to give rise to blood due to SCL deficiency 61. Together, these findings suggest complex and stepwise mechanisms in which cell‐fate specification to endothelium, blood and cardiomyocytes are controlled by positive and negative transcriptional inputs. How ETV2 and SCL each contribute to the repression of cardiomyocyte fate and whether they act in a cascade of events or merely in reinforcing each other activity still remains to be established.

## Transcriptional control of endothelium‐to‐haematopoietic transition

While the generation of HE depends on the program initiated by the ETV2/SCL transcriptional cascade, blood cell emergence from HE is controlled by RUNX1 47, 62. Similar to *Scl*,* Runx1* was initially identified at breakpoints of chromosomal translocations in haematopoietic malignancies 63. Through knockout approaches, RUNX1 was shown to control definitive haematopoiesis emergence (Fig. 1), its deletion only sparing primitive erythrocytes 64, 65, 66. To date, it is not known whether tissue‐resident macrophages of the brain, emerging alongside primitive erythrocytes, depend or not on RUNX1 for their formation. The conditional deletion of *Runx1* at specific stages of development further refined our understanding of its requirement for blood cell emergence. RUNX1 function was shown to be essential in VE‐cadherin or TIE2‐expressing endothelium but no longer required in Vav‐expressing newly formed blood cells 62, 67, pinpointing RUNX1 specific and critical role at the HE stage. Similar to SCL, RUNX1 is also required at later stages of haematopoietic commitment to specific lineages, such as megakaryocytes or T and B cells 68.

**Figure 2:**
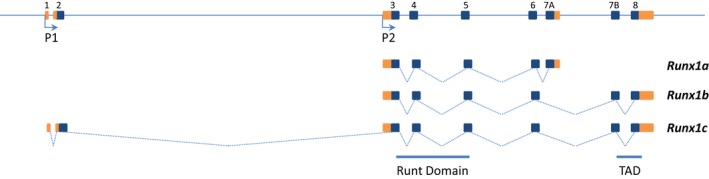
Schematic representation of the mouse *Runx1* locus. Coding exons are represented as blue blocks, untranslated regions as orange blocks. P1, distal promoter; P2, proximal promoter; Runt domain, DNA binding domain; TAD, trans‐activation domain.

The *Runx1* locus encodes several isoforms (*Runx1a*,* b* and *c*) that are differentially expressed from alternative promoters during embryonic development and in adult haematopoiesis (Fig. 2) 69, 70, 71, 72. The proximal promoter P2 controls the transcription of *Runx1a* and *Runx1b* while the distal promoter P1 drives *Runx1c* expression. At the protein level, RUNX1b and RUNX1c only differ by a few amino acids in the N‐terminal region; the functionality of this difference remains unclear. *Runx1a* encodes a truncated version of RUNX1b and may act as an inhibitor of the two other isoforms 73, but little is known about the role of *Runx1a*. Upon enforced expression, this isoform was shown to enhance haematopoiesis 74, 75, 76; additionally, *Runx1a* was shown to be overexpressed in haematological malignancies 77, 78. In the mouse embryo, *Runx1b* is the first isoform expressed in HE prior to the endothelium‐to‐haematopoietic transition; *Runx1c* becomes expressed in newly formed blood progenitors 79. As mentioned above, *Runx1* complete knockout blocks the emergence of all definitive blood cells; in contrast, the specific deletion of the *Runx1c* isoform only marginally affects haematopoiesis 69, 72, 79. Due to the locus structure (Fig. 2), it is not feasible to delete only the *Runx1b* isoform to test its specific requirement in blood cell generation; however, both *Runx1b* and *Runx1c* isoforms are largely functionally interchangeable, as they both equally restore the defects observed in *Runx1* complete knockout embryos 80, 81. It is thought that the timing and expression level of *Runx1b* driven by the proximal promoter are the most critical parameters determining its essential role during endothelial‐to‐haematopoietic transition 71, 82.

RUNX1 transcriptional activity is mediated by a close interaction with its co‐factor CBFβ, which confers increased DNA binding affinity and enhances RUNX1 stability by preventing degradation 83, 84, 85. Transcriptional targets of RUNX1 at the onset of blood specification have been determined using genome‐wide approaches both *in vitro* 51, 86, 87 and *in vivo* 88. At the HE stage, RUNX1 was shown to regulate the expression of genes involved in adhesion and migration, suggesting a unique role of RUNX1 in positioning of the HE population within the vascular compartment 87. Upon endothelial‐to‐haematopoietic transition, RUNX1 was shown to activate the blood program, in collaboration with other transcriptional players such as SCL, GATA2 or FLI1. Genome‐wide analysis of the dynamics of transcription factor binding on target genes revealed that an important function of RUNX1 is to reshape the epigenetic landscape through the assembly of haematopoiesis‐specific binding patterns 89.

Among the many transcriptional targets of RUNX1 identified at this stage of blood cell development, the transcriptional repressors GFI1 and GFI1B hold a special function 86, 90. Through gain‐and‐loss of function approaches both *in vitro* and *in vivo*, it was shown that GFI1 and GFI1B actively downregulate the endothelial program in HE (Fig. 1). Through the recruitment of the chromatin‐modifying protein LSD1, a member of the CoREST repressive complex, GFI1/GFI1B epigenetically silences the endothelial program, allowing the emergence of blood cells. In effect, RUNX1 has a dual activity in repressing the endothelial program while promoting the haematopoietic program. This dual function is highly reminiscent of the functions of ETV2 and SCL in the repression of cardiac specification and concomitant promotion of endothelial and HE programs.

## Endothelial and haematopoietic programs: a balancing act

Specification of the vascular system which entails vasculogenesis and angiogenesis is controlled by key transcription factors of the SOXF, ETS and FOX families 91, 92. Among the SOXF factors, both SOX7 and SOX17 have been implicated in the regulation of HE and early haematopoietic specification. SOX17 was shown to mark HE 93 as well as newly formed HSCs 94 and, upon enforced expression, to maintain HSCs undifferentiated over several passages *in vitro* 95. Similarly, SOX7 was shown to mark HE and upon enforced expression to block the transition from endothelium‐to‐haematopoiesis and to promote HE proliferation 96, 97. Further insight into the molecular mechanism underlying this block revealed that SOX7 physically interacts with RUNX1 in HE and prevents RUNX1 from switching on the blood program and switching off the endothelium program 98. This is thought to create a meta‐stable HE state, poised to give rise to haematopoietic cells upon disruption of the SOX7/RUNX1 interaction. Which signalling pathways or factors disrupt this equilibrium remains to be investigated. Interestingly, SOX17 was also shown to impact RUNX1 activity by actively repressing *Runx1* transcription 99.

From all the studies of blood cell emergence from HE, it is clear that the tight control of RUNX1 activity is one of the most critical parameters. Two recent studies have explored the plasticity of endothelium and its capacity at generating blood cells 100, 101. In both studies, it was shown that during embryonic development, there is a short window of time in which ectopic RUNX1 expression will promote blood cell emergence from non‐HE. Together, these studies suggest that haemogenic competency in endothelial progenitors must be restrained through the active silencing of *Runx1* expression.

## Reprogramming and forward programming for blood cell generation

Understanding the transcriptional network that leads to the formation of haematopoietic stem and progenitor cells (HSPCs) should provide the critical knowledge required for generating these cells *via* reprogramming or forward programming. It is thought that mastering the *de novo* generation of HSPCs from unlimited cell sources (somatic or pluripotent) will provide tailor‐made cell populations usable in the clinic to cure a large range of haematological and autoimmune diseases 102, 103. Many studies have explored the reprograming of somatic cells to blood cells using a variety of transcription factors (Table 1) 104, 105, 106, 107, 108, 109, 110, 111, 112. Similarly, forcing pluripotent stem cells to adopt a HSC or *in vivo* engrafting blood progenitor identity upon differentiation has been achieved *via* forward programming mediated by transcription factors (Table 2) 108, 113, 114, 115, 116. In most studies, the experimental approaches were very similar with lentiviral or retroviral vectors used for expressing potential reprogramming factors in the cell populations of choice. The factors conferring reprogramming were identified from an initial pool of selected transcription factors typically involved in HSC specification or self‐renewal. When large panels of factors were tested, factors effective at reprogramming were identified *via* selective functional assays *in vivo* or *in vitro*. In most studies, the requirement of each identified factor was further determined by individual removal from the reprogramming pool. While all studies reported the generation of blood progenitors as measured by clonogenic replating assays, the generation of cells with long‐term *in vivo* engraftment was by far not as successful. While all the studies reported in Tables 1 and 2 describe the conversion of somatic cells into blood progenitors, the further characterisation of the reprogrammed blood progenitors and their biological output varied quite widely. The study by Pulecio *et al*. 107 described the reprogramming of fibroblasts to unipotent monocyte‐like progenitors. The study performed by Pereira *et al*. 105 showed the generation of blood progenitors with myeloid‐restricted potential. In their study, Szabo *et al*. reprogrammed fibroblasts to progenitors endowed with myeloid and erythroid potential *in vitro* but *in vivo* engraftment was mostly limited to myeloid and no lymphoid potential was observed either *in vitro* or *in vivo* 104. In the study by Batta *et al*. 106, fibroblasts were reprogrammed to blood progenitors with erythroid, myeloid, and lymphoid potential that conferred short‐term *in vivo* engraftment. The only successful reprogramming approaches towards the generation of blood progenitors with long‐term *in vivo* engraftment used starting cell populations with a close relationship to HSC. Riddel *et al*. 109 reprogrammed committed lymphoid and myeloid progenitors to HSCs. Sandler and Lis studies demonstrated long‐term multilineage repopulation (with the exception of T cells for Sandler) using human and murine endothelial cells, respectively, as starting material for reprogramming 110, 112. Sugimura and collaborators obtained long‐term multilineage repopulation by expressing a set of seven transcription factors in haematopoietic cells emerging from HE during the *in vitro* differentiation of human pluripotent stem cells 116. A possible explanation for the successful reprogramming of those cells to HSCs is that endothelium and committed blood progenitors are more amenable to reprogramming towards HSC due to their specific chromatin landscape, likely quite divergent from fibroblast chromatin configuration. To date, it is unknown if the combination of factors used in those studies could reprogram somatic cells more distantly related to the blood lineage.

**Table 1:** Summary of studies reporting the reprogramming of somatic cells to HSPCs.

Reprogrammed population	Reprogramming factors	Expression system	Species	*In vitro* clonogenicity	Long‐term engraftment^a^	References
Fibroblasts	*OCT4*	Lentivirus	Human	Yes	Limited^d^	Szabo *et al*., 2010 104
Fibroblasts	*Gata2, Gfi1b, cFos, Etv6*	Lentiviru Retrovirus	Mouse	Limited^b^	No	Pereira *et al*., 2013 105
Fibroblasts	*Erg, Gata2, Lmo2, Runx1c, Scl*	Lentivirus	Mouse	Yes	No	Batta *et al*., 2014 106
Fibroblasts	*SOX2, miR125b*	Lentiviru Retrovirus	Human	Limited^c^	Not tested	Pulecio *et al*., 2014 107
Fibroblasts	*Scl, Lmo2,Gata2, Ptx2, Sox7,MycN*	Transposon Piggy Bac	Mouse	Yes	Not tested	Vereide *et al*., 2014 108
Committed blood progenitors	*Run1t1, Hlf, Lmo2, Prdm5, Pbx1, Zfp37, Mycn, Meis1*	Lentivirus	Mouse	Yes	Yes	Riddel *et al*., 2014 109
Endothelial cells	*FOSB, GFI1, RUNX1, SPI1*	Lentivirus	Human	Yes	Yes	Sandler *et al*., 2014 110
Fibroblasts	*Scl, Lmo2, Runx1, Bmi1*	Lentiviru Retrovirus	Mouse	Yes	No	Cheng *et al*., 2016 111
Endothelial cells	*Fosb, Gfi1, Runx1, Spi1*	Lentivirus	Mouse	Yes	Yes	Lis *et al*., 2017 112

^a^ Long‐term *in vivo* engraftment means at least 6 months engraftment of all haematopoietic lineages with secondary engraftment. ^b^ Reprogrammed cells only gave rise to very few myeloid colonies in CFU assays. ^c^ Reprogrammed cells only gave rise to monocyte‐like cells. ^d^ Reprogrammed cells gave rise mostly to myeloid with low‐CD45 expression level in primary engraftment and very limited secondary engraftment.

**Table 2:** Summary of studies reporting the forward programming of ESCs to HSPCs.

Reprogrammed population	Reprogramming factors	Expression system	Species	*In vitro* clonogenicity	Long‐term engraftment^a^	References
ESC‐derived cells	*Hoxb4*	Integrated Inducible	Mouse	Yes	Yes	Kyba *et al*., 2004 113
ESC‐derived cells	*Cdx4*	Integrated Inducible	Mouse	Yes	Yes	Wang *et al*., 2005 114
ESC‐derived cells	*ERG, HOXA9, RORA, SOX4, MYB*	Lentivirus	Human	Yes	Short‐term^b^	Doulatov *et al*., 2013 115
ESC‐derived cells	*Scl, Lmo2,Gata2, Pitx2, Sox7,MycN*	Transposon Piggy Bac	Mouse	Yes	Not tested	Vereide *et al*., 2014 108
ESC‐derived cells	*ERG, HOXA5, HOXA9, HOXA10, LCOR, RUNX1, SPI1*	Lentivirus	Human	Yes	Yes	Sugimura *et al*., 2017 116

^a^ Long‐term *in vivo* engraftment means at least 6 months engraftment of all haematopoietic lineages with secondary engraftment. ^b^ Reprogrammed progenitors only provided short‐term *in vivo* engraftment for myeloid and erythroid lineages.

One striking observation, when surveying these studies, is the wide range and variety of transcription factors used for programming (Tables 1 and 2). There is clearly no consensus towards a subset of defined factors promoting blood specification. A few of the listed studies used RUNX1 (5 out of 14 studies) or SCL (4 out of 14 studies), but these two master regulators of blood cell emergence seem dispensable for reprogramming or forward programming. It can be argued, however, that either the reprogrammed population already expressed these factors, as for example in the case of the Riddell study 109, or that *Scl* and *Runx1* are switched on as downstream transcriptional targets of the factors used for reprogramming. An interesting observation is the frequent use of homeotic/homeobox genes as reprogramming factors (5 out of 14 studies), suggesting the important role for re‐patterning, re‐specification or enhanced self‐renewal.

Overall, it seems that the reprogramming of somatic cells to HSPCs does not follow the orderly transcriptional path of developmental haematopoiesis and that multiple combination of factors can promote blood specification in somatic cells. However, one needs to consider that different somatic cell landscapes may require different driving forces to push them towards HSPCs. Additionally, the HSPCs derived from these reprogramming experiments might be qualitatively very different from each other. To date, side‐by‐side comparisons of reprogramming protocols have not been published. Forward programming of pluripotent stem cells represent a different challenge as this consists in pushing undifferentiated cells towards a lineage of choice. It is noteworthy that most of these studies employ homeotic genes to drive forward programming. While great progress has been made towards the *de novo* generation of HSPCs *via* reprogramming, it remains uncertain whether these protocols can be translated to the clinic, given the expression of potential harmful factors (some of them known to confer leukaemias) or the random genomic insertion of exogenous factors that may activate harmful genes.

## Conclusion and perspective

We have come a long way in understanding how blood cell emergence is controlled at the molecular level and what are the main players in this developmental process. However, there are still many unanswered questions: what differentiates HE from non‐HE at the molecular level? How is the level of RUNX1 regulated during the transition from HE to haematopoietic cells? Are there different types of HE, is the microenvironment critical in conferring specificity to HE or is it a combination of both? Will we be able to use all this basic knowledge towards the clinic for the benefit of patients?
